# (1-Naphthyl­imino­meth­yl)ferrocene

**DOI:** 10.1107/S1600536808026330

**Published:** 2008-08-20

**Authors:** Yunbo Zang

**Affiliations:** a Department of Chemistry, Shangqiu Normal College, Shangqiu, Henan 476000, People’s Republic of China

## Abstract

In the title mol­ecule, [Fe(C_5_H_5_)(C_16_H_12_N)], the cyclo­penta­dienyl rings are approximately eclipsed and the inter­planar angle is 0.8 (7)°. The Fe atom is slightly closer to the substituted cyclo­penta­dienyl ring, with an Fe⋯centroid distance of 1.639 (2) Å, compared with 1.645 (2) Å for the unsubstituted ring. The C=N double bond is essentially coplanar with the substituted cyclo­penta­dienyl ring with a deviation of 10.3 (1)°. The angle formed by the C=N double bond and the naphthal­ene ring system is 47.1 (1)°. The C—N=C—C torsion angle is 177.32 (5)°.

## Related literature

For related crystal structures, see: Kovac *et al.* (2004 ▶). For related literature, see: Baar *et al.* (2000 ▶); Johnson & Sames (2000 ▶); Staveren & Metzler-Nolte (2004 ▶).
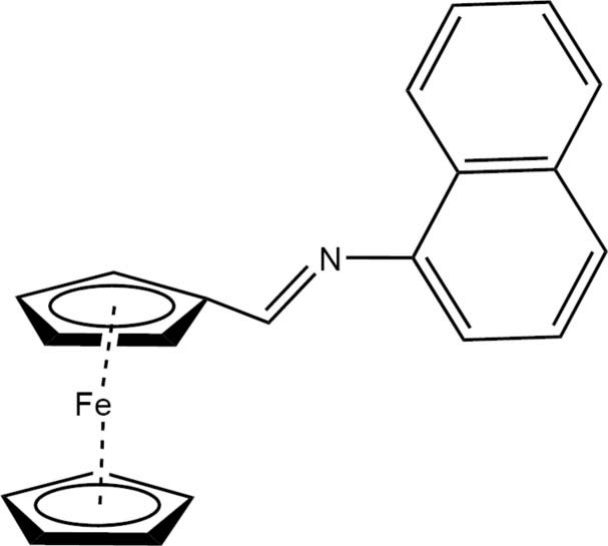


## Experimental

### 

#### Crystal data

[Fe(C_5_H_5_)(C_16_H_12_N)]
*M* *_r_* = 339.21
Monoclinic, 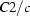

*a* = 19.5283 (4) Å
*b* = 7.3578 (2) Å
*c* = 23.7390 (5) Å
β = 108.8260 (10)°
*V* = 3228.47 (13) Å^3^
*Z* = 8
Mo *K*α radiation
μ = 0.93 mm^−1^
*T* = 293 (2) K
0.24 × 0.20 × 0.15 mm

#### Data collection

Bruker SMART 1000 CCD diffractometer
Absorption correction: multi-scan (*SADABS*; Sheldrick, 1996 ▶) *T*_min_ = 0.807, *T*_max_ = 0.873
18034 measured reflections
3164 independent reflections
2740 reflections with *I* > 2σ(*I*)
*R*_int_ = 0.022

#### Refinement

*R*[*F* ^2^ > 2σ(*F* ^2^)] = 0.028
*wR*(*F* ^2^) = 0.078
*S* = 1.05
3164 reflections
209 parameters
H-atom parameters constrained
Δρ_max_ = 0.19 e Å^−3^
Δρ_min_ = −0.19 e Å^−3^

### 

Data collection: *SMART* (Bruker, 1997 ▶); cell refinement: *SAINT* (Bruker, 1997 ▶); data reduction: *SAINT*; program(s) used to solve structure: *SHELXS97* (Sheldrick, 2008 ▶); program(s) used to refine structure: *SHELXL97* (Sheldrick, 2008 ▶); molecular graphics: *SHELXTL* (Sheldrick, 2008 ▶); software used to prepare material for publication: *SHELXTL*.

## Supplementary Material

Crystal structure: contains datablock(s) I, global. DOI: 10.1107/S1600536808026330/lh2673sup1.cif

Structure factors: contains datablock(s) I. DOI: 10.1107/S1600536808026330/lh2673Isup2.hkl

Additional supplementary materials:  crystallographic information; 3D view; checkCIF report

## Figures and Tables

**Fig. 1.**
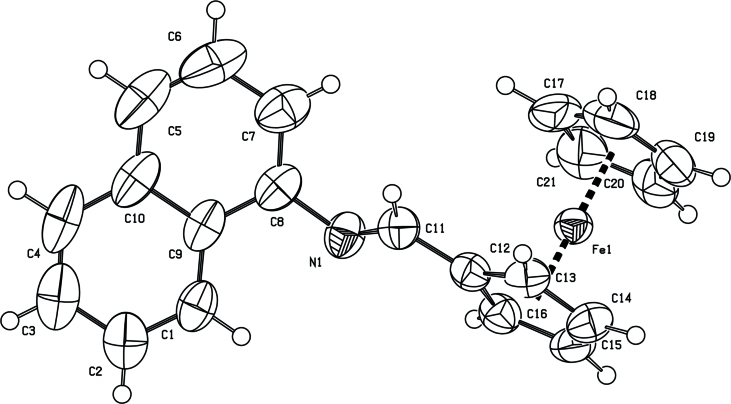
Molecular structure of title compound. Displacement ellipsoids are drawn at the 30% probability for non-H atoms.

## supplementary crystallographic information

### Comment

Transition metal complexes derived from ferrocene have attracted great intrest due to their applications as precursors for the synthesis of organic as well as organometallic compounds (Johnson & Sames, 2000), in homogeneous catalysis (Baar *et al.*, 2000), or even in biological chemistry (Staveren & Metzler-Nolte, 2004). In this paper we reported the synthesis and crystal structure of the title compound (1). The molecular structure of (I) [Fig. 1] consists of a naphthyliminomethyl moiety and a ferrocene unit. In the ferrocene unit, the unsubstituted cyclopentadienyl ring and the substituted cyclopentadienyl ring are approximately eclipsed, and the interplannar angle is 0.8 (7)°. The Fe atom is slightly closer to the substituted cyclopentadienyl ring with a Fe···ring centroid distance of 1.639 (2) vs. 1.645 (2) for the other ring. The C=N double bond is almost parallel to the substituted cyclopentadienyl ring with the deviation 10.3 (1)°. The angle formed by the C=N double bond and the naphthyl ring is 47.1 (1) °. The torsion angle C-N=C-C is 177.32 (5)°.

### Experimental

Ferrocenecarbaldehyde (1.2 g, 2.79 mmol) was dissolved in 30 ml benzene at room temperature, after the material had dissolved completely, 0.8 g naphthylamine (5.58 mmol) was added to the solution. The mixture was refluxed with a Dean-Stark apparatus to remove the water produced during the reaction. After 5 h, the solvents were removed on a rotary evaporator and the residue was recrystallized in ether to give orange crystals 1.55 g. Yield 82%.

### Refinement

H atoms were positioned geometrically and refined using a riding model with C—H = 0.93–0.98 Å and with *U*_iso_(H) = 1.2 times *U*_eq_(C).

### Crystal data

**Table:**

[Fe(C_5_H_5_)(C_16_H_12_N)]	*F*(000) = 1408
*M**_r_* = 339.21	*D*_x_ = 1.396 Mg m^−^^3^
Monoclinic, *C*2/*c*	Mo *K*α radiation, λ = 0.71073 Å
Hall symbol: -C 2yc	Cell parameters from 8631 reflections
*a* = 19.5283 (4) Å	θ = 2.2–26.9°
*b* = 7.3578 (2) Å	µ = 0.93 mm^−^^1^
*c* = 23.7390 (5) Å	*T* = 293 K
β = 108.826 (1)°	Block, orange
*V* = 3228.47 (13) Å^3^	0.24 × 0.20 × 0.15 mm
*Z* = 8	

### Data collection

**Table:**

Bruker SMART 1000 CCD diffractometer	3164 independent reflections
Radiation source: fine-focus sealed tube	2740 reflections with *I* > 2σ(*I*)
graphite	*R*_int_ = 0.022
φ and ω scans	θ_max_ = 26.0°, θ_min_ = 1.8°
Absorption correction: multi-scan (*SADABS*; Sheldrick, 1996)	*h* = −24→24
*T*_min_ = 0.807, *T*_max_ = 0.873	*k* = −9→8
18034 measured reflections	*l* = −29→29

### Refinement

**Table:**

Refinement on *F*^2^	Secondary atom site location: difference Fourier map
Least-squares matrix: full	Hydrogen site location: inferred from neighbouring sites
*R*[*F*^2^ > 2σ(*F*^2^)] = 0.028	H-atom parameters constrained
*wR*(*F*^2^) = 0.078	*w* = 1/[σ^2^(*F*_o_^2^) + (0.0401*P*)^2^ + 1.5179*P*] where *P* = (*F*_o_^2^ + 2*F*_c_^2^)/3
*S* = 1.05	(Δ/σ)_max_ = 0.001
3164 reflections	Δρ_max_ = 0.19 e Å^−^^3^
209 parameters	Δρ_min_ = −0.19 e Å^−^^3^
0 restraints	Extinction correction: *SHELXL97* (Sheldrick, 2008), Fc^*^=kFc[1+0.001xFc^2^λ^3^/sin(2θ)]^-1/4^
Primary atom site location: structure-invariant direct methods	Extinction coefficient: 0.00052 (11)

### Special details

**Table:**

Geometry. All e.s.d.'s (except the e.s.d. in the dihedral angle between two l.s. planes) are estimated using the full covariance matrix. The cell e.s.d.'s are taken into account individually in the estimation of e.s.d.'s in distances, angles and torsion angles; correlations between e.s.d.'s in cell parameters are only used when they are defined by crystal symmetry. An approximate (isotropic) treatment of cell e.s.d.'s is used for estimating e.s.d.'s involving l.s. planes.
Refinement. Refinement of *F*^2^ against ALL reflections. The weighted *R*-factor *wR* and goodness of fit *S* are based on *F*^2^, conventional *R*-factors *R* are based on *F*, with *F* set to zero for negative *F*^2^. The threshold expression of *F*^2^ > σ(*F*^2^) is used only for calculating *R*-factors(gt) *etc*. and is not relevant to the choice of reflections for refinement. *R*-factors based on *F*^2^ are statistically about twice as large as those based on *F*, and *R*- factors based on ALL data will be even larger.

### Fractional atomic coordinates and isotropic or equivalent isotropic displacement parameters (Å^2^)

**Table:**

	*x*	*y*	*z*	*U*_iso_*/*U*_eq_	
Fe1	0.244742 (12)	0.85825 (3)	0.123387 (10)	0.04360 (11)	
N1	0.38867 (8)	0.9603 (2)	0.28006 (7)	0.0572 (4)	
C1	0.48453 (10)	0.8798 (3)	0.39478 (9)	0.0601 (5)	
H1	0.4528	0.7910	0.3734	0.072*	
C2	0.53061 (11)	0.8400 (4)	0.45027 (10)	0.0738 (6)	
H2	0.5295	0.7251	0.4663	0.089*	
C3	0.57918 (12)	0.9697 (5)	0.48302 (11)	0.0864 (8)	
H3	0.6108	0.9408	0.5206	0.104*	
C4	0.58042 (11)	1.1370 (4)	0.46033 (12)	0.0839 (8)	
H4	0.6129	1.2226	0.4828	0.101*	
C5	0.53241 (16)	1.3586 (4)	0.37809 (14)	0.0928 (9)	
H5	0.5645	1.4463	0.3997	0.111*	
C6	0.48643 (18)	1.4021 (4)	0.32367 (15)	0.0979 (9)	
H6	0.4867	1.5187	0.3086	0.118*	
C7	0.43728 (14)	1.2685 (3)	0.28922 (11)	0.0792 (6)	
H7	0.4055	1.2983	0.2518	0.095*	
C8	0.43708 (10)	1.0975 (3)	0.31132 (9)	0.0592 (5)	
C9	0.48424 (9)	1.0520 (3)	0.36948 (9)	0.0566 (5)	
C10	0.53325 (11)	1.1864 (3)	0.40271 (11)	0.0706 (6)	
C11	0.38579 (9)	0.9244 (3)	0.22709 (8)	0.0521 (4)	
H11	0.4169	0.9852	0.2110	0.063*	
C12	0.33590 (9)	0.7921 (3)	0.19079 (7)	0.0478 (4)	
C13	0.33816 (9)	0.7214 (3)	0.13539 (8)	0.0522 (4)	
H13	0.3747	0.7495	0.1165	0.063*	
C14	0.27871 (11)	0.6024 (3)	0.11281 (9)	0.0582 (5)	
H14	0.2666	0.5353	0.0752	0.070*	
C15	0.23897 (11)	0.5992 (2)	0.15338 (9)	0.0559 (5)	
H15	0.1945	0.5302	0.1485	0.067*	
C16	0.27343 (9)	0.7156 (3)	0.20106 (8)	0.0527 (4)	
H16	0.2573	0.7409	0.2353	0.063*	
C17	0.23857 (15)	1.1334 (3)	0.11874 (14)	0.0953 (10)	
H17	0.2738	1.2181	0.1443	0.114*	
C18	0.24075 (12)	1.0616 (3)	0.06452 (12)	0.0833 (7)	
H18	0.2775	1.0878	0.0456	0.100*	
C19	0.18066 (11)	0.9451 (3)	0.04201 (9)	0.0702 (6)	
H19	0.1680	0.8761	0.0047	0.084*	
C20	0.14173 (10)	0.9477 (3)	0.08299 (10)	0.0702 (6)	
H20	0.0976	0.8786	0.0792	0.084*	
C21	0.17731 (13)	1.0623 (3)	0.13038 (12)	0.0843 (7)	
H21	0.1623	1.0891	0.1651	0.101*	

### Atomic displacement parameters (Å^2^)

**Table:**

	*U*^11^	*U*^22^	*U*^33^	*U*^12^	*U*^13^	*U*^23^
Fe1	0.04306 (15)	0.03924 (16)	0.04794 (16)	0.00129 (10)	0.01389 (11)	0.00106 (10)
N1	0.0501 (8)	0.0651 (11)	0.0572 (9)	−0.0100 (7)	0.0186 (7)	−0.0072 (8)
C1	0.0467 (9)	0.0704 (14)	0.0648 (12)	−0.0069 (9)	0.0201 (9)	−0.0205 (10)
C2	0.0598 (12)	0.0917 (18)	0.0681 (13)	0.0069 (11)	0.0181 (11)	−0.0153 (12)
C3	0.0578 (12)	0.120 (2)	0.0766 (15)	0.0029 (14)	0.0148 (11)	−0.0353 (16)
C4	0.0484 (11)	0.117 (2)	0.0868 (17)	−0.0228 (13)	0.0227 (11)	−0.0562 (16)
C5	0.0972 (19)	0.090 (2)	0.109 (2)	−0.0442 (15)	0.0583 (18)	−0.0454 (17)
C6	0.135 (3)	0.0633 (16)	0.125 (2)	−0.0290 (16)	0.082 (2)	−0.0183 (16)
C7	0.0958 (17)	0.0693 (16)	0.0859 (16)	−0.0127 (13)	0.0480 (14)	−0.0083 (13)
C8	0.0545 (10)	0.0604 (13)	0.0720 (12)	−0.0114 (9)	0.0333 (10)	−0.0141 (10)
C9	0.0427 (9)	0.0695 (13)	0.0647 (11)	−0.0123 (9)	0.0271 (8)	−0.0253 (10)
C10	0.0572 (11)	0.0777 (16)	0.0901 (16)	−0.0231 (11)	0.0421 (12)	−0.0372 (13)
C11	0.0422 (8)	0.0560 (11)	0.0589 (10)	0.0003 (8)	0.0174 (8)	0.0008 (9)
C12	0.0443 (8)	0.0480 (10)	0.0505 (9)	0.0060 (8)	0.0144 (7)	0.0054 (8)
C13	0.0516 (9)	0.0475 (11)	0.0619 (11)	0.0077 (8)	0.0247 (8)	−0.0005 (9)
C14	0.0682 (12)	0.0426 (10)	0.0664 (12)	0.0032 (9)	0.0253 (10)	−0.0086 (9)
C15	0.0606 (10)	0.0410 (10)	0.0665 (12)	−0.0043 (8)	0.0211 (9)	0.0063 (9)
C16	0.0544 (10)	0.0558 (11)	0.0497 (9)	−0.0005 (9)	0.0190 (8)	0.0075 (8)
C17	0.0882 (19)	0.0363 (13)	0.131 (3)	0.0101 (11)	−0.0070 (18)	0.0084 (13)
C18	0.0686 (13)	0.0715 (16)	0.1028 (19)	0.0034 (12)	0.0181 (13)	0.0452 (15)
C19	0.0632 (12)	0.0800 (16)	0.0589 (12)	0.0044 (11)	0.0078 (9)	0.0219 (11)
C20	0.0472 (10)	0.0786 (16)	0.0771 (14)	0.0138 (10)	0.0092 (10)	0.0123 (12)
C21	0.0770 (15)	0.0724 (16)	0.0928 (17)	0.0347 (13)	0.0126 (13)	−0.0089 (14)

### Geometric parameters (Å, °)

**Table:**

Fe1—C13	2.0219 (17)	C7—C8	1.364 (3)
Fe1—C17	2.029 (2)	C7—H7	0.9300
Fe1—C18	2.031 (2)	C8—C9	1.430 (3)
Fe1—C12	2.0317 (16)	C9—C10	1.424 (3)
Fe1—C19	2.0362 (19)	C11—C12	1.448 (3)
Fe1—C16	2.0366 (17)	C11—H11	0.9300
Fe1—C14	2.0379 (18)	C12—C13	1.428 (2)
Fe1—C20	2.0383 (19)	C12—C16	1.434 (2)
Fe1—C21	2.039 (2)	C13—C14	1.414 (3)
Fe1—C15	2.0506 (18)	C13—H13	0.9800
N1—C11	1.269 (2)	C14—C15	1.419 (3)
N1—C8	1.418 (2)	C14—H14	0.9800
C1—C2	1.366 (3)	C15—C16	1.406 (3)
C1—C9	1.402 (3)	C15—H15	0.9800
C1—H1	0.9300	C16—H16	0.9800
C2—C3	1.393 (3)	C17—C18	1.405 (4)
C2—H2	0.9300	C17—C21	1.412 (4)
C3—C4	1.347 (4)	C17—H17	0.9800
C3—H3	0.9300	C18—C19	1.412 (3)
C4—C10	1.428 (4)	C18—H18	0.9800
C4—H4	0.9300	C19—C20	1.415 (3)
C5—C6	1.352 (4)	C19—H19	0.9800
C5—C10	1.393 (4)	C20—C21	1.398 (3)
C5—H5	0.9300	C20—H20	0.9800
C6—C7	1.432 (4)	C21—H21	0.9800
C6—H6	0.9300		
			
C13—Fe1—C17	122.66 (10)	C1—C9—C10	118.8 (2)
C13—Fe1—C18	107.24 (9)	C1—C9—C8	122.50 (18)
C17—Fe1—C18	40.48 (10)	C10—C9—C8	118.7 (2)
C13—Fe1—C12	41.27 (7)	C5—C10—C9	119.0 (2)
C17—Fe1—C12	107.65 (9)	C5—C10—C4	123.4 (2)
C18—Fe1—C12	123.20 (8)	C9—C10—C4	117.7 (2)
C13—Fe1—C19	122.71 (8)	N1—C11—C12	122.42 (17)
C17—Fe1—C19	68.15 (11)	N1—C11—H11	118.8
C18—Fe1—C19	40.61 (9)	C12—C11—H11	118.8
C12—Fe1—C19	159.46 (8)	C13—C12—C16	107.11 (16)
C13—Fe1—C16	69.13 (7)	C13—C12—C11	125.31 (16)
C17—Fe1—C16	123.88 (11)	C16—C12—C11	127.49 (16)
C18—Fe1—C16	160.13 (10)	C13—C12—Fe1	69.00 (10)
C12—Fe1—C16	41.27 (7)	C16—C12—Fe1	69.55 (10)
C19—Fe1—C16	157.80 (8)	C11—C12—Fe1	123.92 (13)
C13—Fe1—C14	40.77 (8)	C14—C13—C12	107.99 (16)
C17—Fe1—C14	158.57 (11)	C14—C13—Fe1	70.23 (10)
C18—Fe1—C14	122.49 (11)	C12—C13—Fe1	69.73 (9)
C12—Fe1—C14	68.81 (8)	C14—C13—H13	126.0
C19—Fe1—C14	107.33 (10)	C12—C13—H13	126.0
C16—Fe1—C14	68.30 (8)	Fe1—C13—H13	126.0
C13—Fe1—C20	159.35 (9)	C13—C14—C15	108.38 (17)
C17—Fe1—C20	67.66 (10)	C13—C14—Fe1	69.01 (10)
C18—Fe1—C20	68.01 (9)	C15—C14—Fe1	70.18 (10)
C12—Fe1—C20	158.25 (8)	C13—C14—H14	125.8
C19—Fe1—C20	40.63 (8)	C15—C14—H14	125.8
C16—Fe1—C20	122.26 (8)	Fe1—C14—H14	125.8
C14—Fe1—C20	123.46 (9)	C16—C15—C14	108.16 (16)
C13—Fe1—C21	158.81 (9)	C16—C15—Fe1	69.35 (10)
C17—Fe1—C21	40.62 (11)	C14—C15—Fe1	69.22 (10)
C18—Fe1—C21	68.36 (11)	C16—C15—H15	125.9
C12—Fe1—C21	122.47 (9)	C14—C15—H15	125.9
C19—Fe1—C21	68.33 (10)	Fe1—C15—H15	125.9
C16—Fe1—C21	107.57 (10)	C15—C16—C12	108.36 (16)
C14—Fe1—C21	159.17 (10)	C15—C16—Fe1	70.42 (10)
C20—Fe1—C21	40.11 (9)	C12—C16—Fe1	69.18 (9)
C13—Fe1—C15	68.67 (8)	C15—C16—H16	125.8
C17—Fe1—C15	159.62 (11)	C12—C16—H16	125.8
C18—Fe1—C15	158.31 (11)	Fe1—C16—H16	125.8
C12—Fe1—C15	68.67 (8)	C18—C17—C21	108.5 (2)
C19—Fe1—C15	122.37 (9)	C18—C17—Fe1	69.86 (13)
C16—Fe1—C15	40.24 (8)	C21—C17—Fe1	70.08 (13)
C14—Fe1—C15	40.60 (8)	C18—C17—H17	125.7
C20—Fe1—C15	107.88 (9)	C21—C17—H17	125.7
C21—Fe1—C15	123.16 (10)	Fe1—C17—H17	125.7
C11—N1—C8	118.44 (16)	C17—C18—C19	107.9 (2)
C2—C1—C9	121.2 (2)	C17—C18—Fe1	69.66 (13)
C2—C1—H1	119.4	C19—C18—Fe1	69.88 (12)
C9—C1—H1	119.4	C17—C18—H18	126.0
C1—C2—C3	120.6 (3)	C19—C18—H18	126.0
C1—C2—H2	119.7	Fe1—C18—H18	126.0
C3—C2—H2	119.7	C18—C19—C20	107.3 (2)
C4—C3—C2	120.0 (2)	C18—C19—Fe1	69.51 (12)
C4—C3—H3	120.0	C20—C19—Fe1	69.76 (11)
C2—C3—H3	120.0	C18—C19—H19	126.4
C3—C4—C10	121.8 (2)	C20—C19—H19	126.4
C3—C4—H4	119.1	Fe1—C19—H19	126.4
C10—C4—H4	119.1	C21—C20—C19	108.9 (2)
C6—C5—C10	122.0 (2)	C21—C20—Fe1	69.98 (12)
C6—C5—H5	119.0	C19—C20—Fe1	69.60 (11)
C10—C5—H5	119.0	C21—C20—H20	125.5
C5—C6—C7	120.0 (3)	C19—C20—H20	125.5
C5—C6—H6	120.0	Fe1—C20—H20	125.5
C7—C6—H6	120.0	C20—C21—C17	107.3 (2)
C8—C7—C6	119.9 (3)	C20—C21—Fe1	69.91 (12)
C8—C7—H7	120.1	C17—C21—Fe1	69.30 (13)
C6—C7—H7	120.1	C20—C21—H21	126.3
C7—C8—N1	122.6 (2)	C17—C21—H21	126.3
C7—C8—C9	120.4 (2)	Fe1—C21—H21	126.3
N1—C8—C9	116.87 (18)		
